# Resveratrol affects Zika virus replication *in vitro*

**DOI:** 10.1038/s41598-019-50674-3

**Published:** 2019-10-04

**Authors:** Azirah Mohd, Nurhafiza Zainal, Kim-Kee Tan, Sazaly AbuBakar

**Affiliations:** 1 Tropical Infectious Diseases Research and Education Centre (TIDREC), University of Malaya, Kuala Lumpur, Malaysia; 2 Department of Medical Microbiology, University of Malaya, Kuala Lumpur, Malaysia; 3 Faculty of Medicine, University of Malaya, Kuala Lumpur, Malaysia

**Keywords:** Drug development, Infection

## Abstract

Zika virus (ZIKV) infection is a serious public health concern. ZIKV infection has been associated with increased occurrences of microcephaly among newborns and incidences of Guillain-Barré syndrome among adults. No specific therapeutics or vaccines are currently available to treat and protect against ZIKV infection. Here, a plant-secreted phytoalexin, resveratrol (RES), was investigated for its ability to inhibit ZIKV replication *in vitro*. Several RES treatment regimens were used. The ZIKV titers of mock- and RES-treated infected cell cultures were determined using the focus-forming assay and the Zika mRNA copy number as determined using qRT-PCR. Our results suggested that RES treatment reduced ZIKV titers in a dose-dependent manner. A reduction of >90% of virus titer and ZIKV mRNA copy number was achieved when infected cells were treated with 80 µM of RES post-infection. Pre-incubation of the virus with 80 µM RES showed >30% reduction in ZIKV titers and ZIKV mRNA copy number, implying potential direct virucidal effects of RES against the virus. The RES treatment reduced >70% virus titer in the anti-adsorption assay, suggesting the possibility that RES also interferes with ZIKV binding. However, there was no significant decrease in ZIKV titer when a short-period of RES treatment was applied to cells before ZIKV infection (pre-infection) and after the virus bound to the cells (virus internalization inhibition), implying that RES acts through its continuous presence in the cell cultures after virus infection. Overall, our results suggested that RES exhibited direct virucidal activity against ZIKV and possessed anti-ZIKV replication properties, highlighting the need for further exploration of RES as a potential antiviral molecule against ZIKV infection.

## Introduction

Zika virus (ZIKV), a positive single-stranded RNA virus, belongs to the *Flaviviridae* family of the genus *Flavivirus*. The virus was first isolated in Uganda in 1947 from monkeys. Initially, the virus is known to cause isolated outbreaks in Asia and Africa^1,2^. The first large outbreak of ZIKV, however, occurred on Yap Island, the Federated States of Micronesia in 2007^3^, followed by another massive outbreak in French Polynesia from the year 2013 to 2015^4^. More recently, from 2015 to 2016, ZIKV epidemic occurred throughout South America, Central America and the Caribbean^5^. The recent ZIKV infections in Asia have been reported mostly in China, India, Indonesia, Malaysia, Philippines, Singapore and Taiwan^6^. Recent phylogenetic analysis using nucleotide sequences derived from the envelope (E) and non-structural protein 5 (NS5), revealed three ZIKV lineages; Asian, African I and African II^7^. In general, ZIKV is transmitted to human via bites of an infected female mosquito belonging to the *Culicidae* family of the genus *Aedes*, specifically *Aedes aegypti*^8^. The non-vectorial transmission of ZIKV which include sexual transmission^9,10^, perinatal transmission^11^ and blood transfusion^12^ were recently recognized.

Early clinical symptoms of ZIKV infection, which include fever and headache, are quite similar to symptoms of other flavivirus infections, including dengue and chikungunya^13^. Other common symptoms of ZIKV infection include arthralgia, extremity edema, conjunctivitis, retro-orbital pain, and maculopapular rashes, which usually dispersed from the face to the limbs^14,15^. In the more serious ZIKV infection, neurological complications such as microcephaly in the newborns and Guillain-Barré syndrome (GBS) in adults have been reported^16,17^. The recent large outbreak of ZIKV infection in the Americas, especially in Brazil, which was accompanied by unprecedented incidents of microcephalic babies born to ZIKV-infected mothers, triggered WHO to declare ZIKV infection as public health emergency of international concern (PHEIC). With no vaccine available, there is an urgent need to find a potentially effective therapeutic against the virus. A number of potential antivirals have been identified for a number of flaviviruses^18–21^. These include several natural compounds such as flavonoids^22,23^, polysaccharide^24^, alkaloid^25^, terpenoid^26^ and phenolic^27^. Resveratrol (3, 5, 4′ –trihydroxy-trans-stilbene, RES), a stilbenoid, derived mainly from grapes and peanuts^28^ was shown in earlier studies to exert potential beneficial effects against human and animal viruses^18,29–33^. A recent structure-based study showed two RES analogs could possess antiviral activities against ZIKV NS2B_18_NS3 and helicase^34^. Here, we determined the effects of RES treatment *in vitro* on ZIKV replication.

## Results

### Cytotoxicity of RES against Huh7 cells and Vero cells

**Figure 1:**
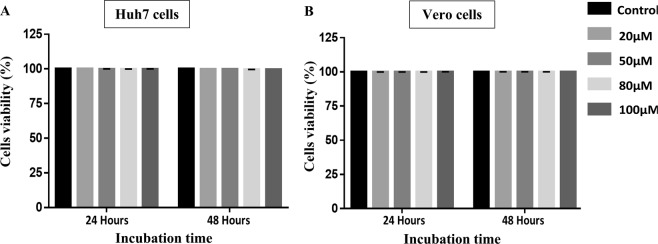
Cytotoxic effects of RES on Huh7 cells and Vero cells. The cytotoxicity of RES was determined using the MTS assay. Huh7 cells (**A**) and Vero cells (**B**) were mock-treated or treated with RES at the concentrations of 20 µM, 50 µM, 80 µM, and 100 µM for 24 or 48 h. The experiments were performed in triplicates and the data obtained were analyzed using Graph Pad Prism 7 (Graph Pad Software Inc., San Diego, CA, USA, 2016).

The MTS cytotoxicity assay was used to determine the potential toxicity of RES against Huh7 cells and Vero cells. Both cells were treated with increasing concentrations of RES; 20 µM, 50 µM, 80 µM, and 100 µM and incubated for 24 or 48 h prior to MTS cytotoxicity evaluation. The viability of the RES-treated cells was determined and the viability cells count of the diluent-treated (mock-treated) cells was used as the control. RES treatment of Huh7 cells at the concentrations of 20 µM, 50 µM, 80 µM, and 100 µM for 24 and 48 h resulted in cell viability of more than 99% at all concentrations used (Fig. 1A). Comparably, RES treatment of Vero cells for 24 and 48 h at concentrations of 20 µM, 50 µM, 80 µM, and 100 µM also resulted in more than 99% cell viability (Fig. 1B). These results suggested that the cell viability of both Huh7 and Vero cells remained at more than 99% following treatments with up to 100 µM of RES for both 24 and 48 h treatment regimens, suggesting that RES did not cause significant cytotoxicity to Huh7 and Vero cells.

### The inhibitory effects of RES against ZIKV

**Figure 2:**
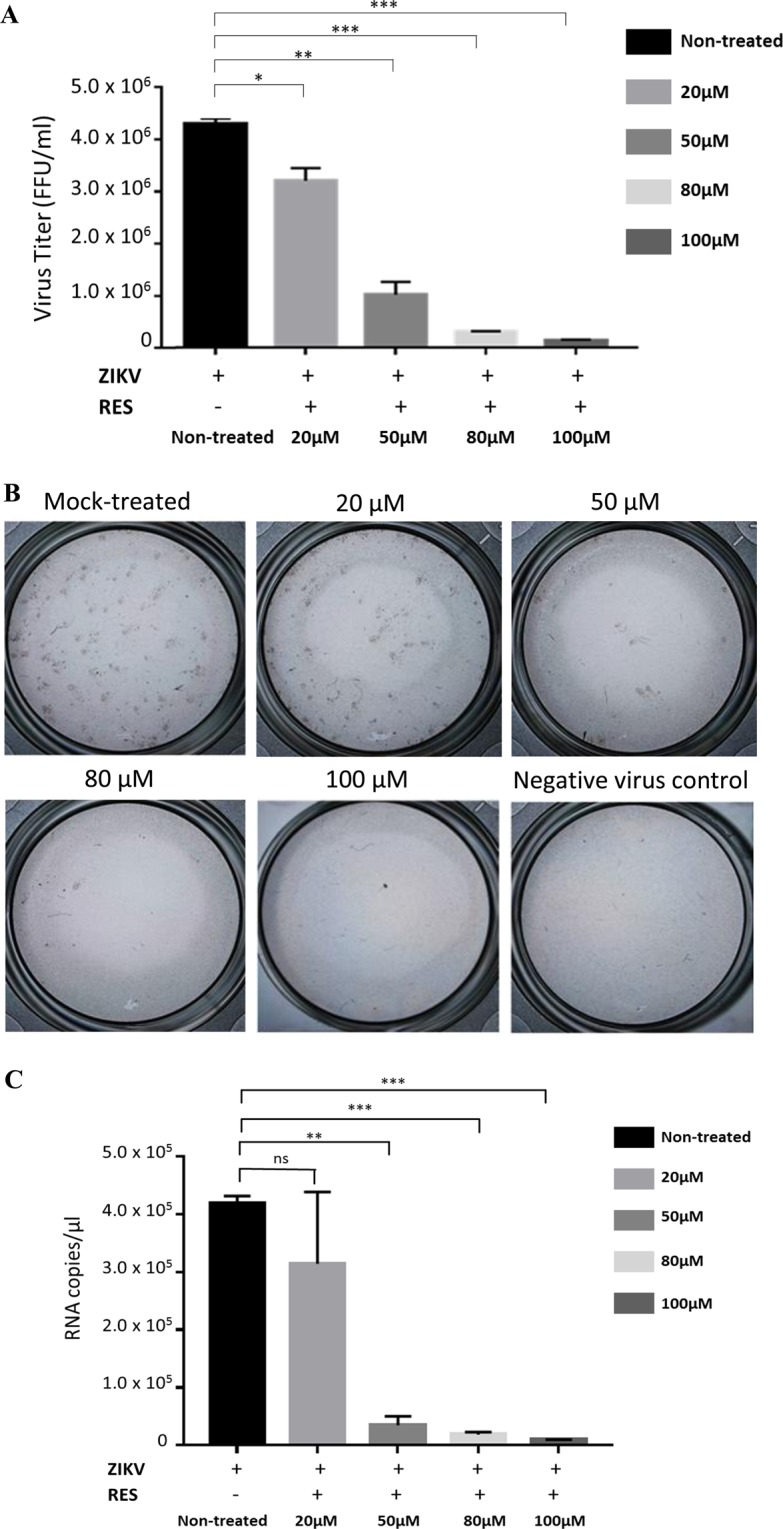
Antiviral activity of RES against ZIKV. Huh7 cells were treated with different concentrations of RES before and after ZIKV infection at an MOI of 1 for 48 h. The cell supernatant was collected and virus titer was determined using the focus-forming assay (**A**). Foci formed on mock-treated and RES-treated cells (20 µM, 50 µM, 80 µM or with 100 µM) after three days of incubation were shown. The mock-treated and non-infected cells were used as controls. (**B**). Real-time PCR was performed to quantify the ZIKV mRNA copy numbers (**C**). Data obtained from duplicate assays were analyzed and plotted using Graph Pad Prism 7 (Graph Pad Software Inc., San Diego, CA, USA, 2016). Statistical differences between groups were as follow: *P < 0.05, **P < 0.01, ***P < 0.001.

In order to investigate the antiviral effects of RES against ZIKV, we determined the virus replication inhibition in cells treated with different concentrations of RES, while the mock-treated cells were used as control. Huh7 cells were mock-treated or treated with different concentrations of RES (20 µM, 50 µM, 80 µM, and 100 µM) prior and post ZIKV infection at an MOI of 1. After 48 h, the supernatants of the non- and RES-treated cell cultures were harvested. The supernatants were used to infect Vero cells for the focus-forming assay and the qRT-PCR assay. Treatment of cells with 20 µM, 50 µM, 80 µM and 100 µM of RES reduced the number of foci formed by 25%, 76%, 93% (1 log) and 97% (1 log), respectively (Fig. 2A,B). Correspondingly, ZIKV mRNA copy numbers were significantly reduced by the RES treatment (20 µM; 25% virus reduction, 50 µM; 92% [1 log] virus reduction, 80 µM; 96% [1 log] virus reduction, 100 µM; 98% [2 log] virus reduction) (Fig. 2C), suggesting the inhibitory effects of RES against ZIKV replication.

### RES exhibited its antiviral effects after ZIKV entry

**Figure 3:**
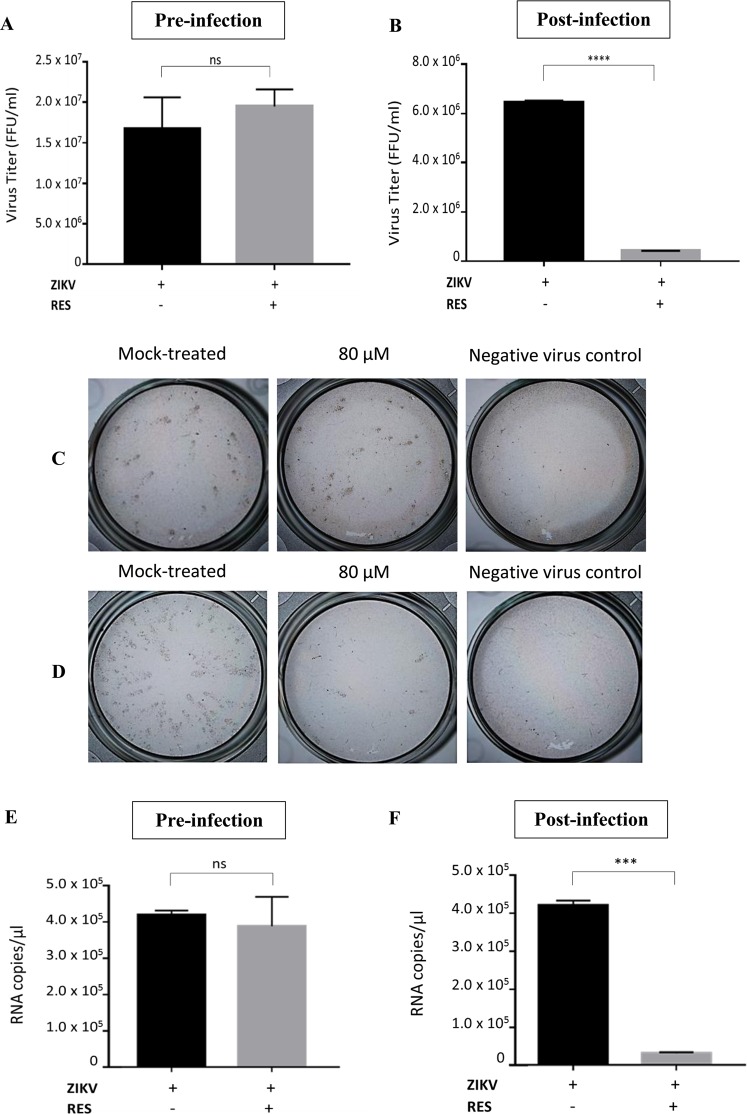
Inhibitory effects of RES after virus entry. Huh7 cells were treated with 80 µM RES before (pre-infection, **A**) or after (post-infection, **B**) ZIKV infection at an MOI of 1 for 48 h. The cell supernatants were collected after 48 h and virus titer was determined using focus-forming assay. Foci formed in mock-treated, pre-infection (**C**) and post-infection (**D**) conditions after three days of incubation were shown. The mock-treated and non-infected cells were used as controls. The ZIKV mRNA copy numbers in pre-infection (**E**) and post-infection (**F**) were quantified using qRT-PCR. Data obtained from duplicate assays were analyzed and plotted using Graph Pad Prism 7 (Graph Pad Software Inc., San Diego, CA, USA, 2016). Statistical differences between groups were as follow: ***P < 0.001, ****P < 0.0001.

To obtain insight into the potential antiviral mechanisms of RES, we examined the possible mode of actions of the phytoalexin. Huh7 cells were treated with 80 µM of RES prior (pre-infection) or after (post-infection) ZIKV infection at an MOI of 1. After 48 h incubation, the supernatants were harvested from the mock- and RES-treated Huh7 cells. Subsequently, harvested supernatants were added onto the Vero cells for the virus titer evaluation using the foci forming assay. In addition, the virus mRNA copy number was also determined using qRT-PCR. The pre-infection treatment regimen, where the cells were treated with RES before the virus infection, showed 5% increased of ZIKV titers than the mock-treated cells (Fig. 3A,C), implying no significant reduction of the virus titer. However, post-infection treatment regimen, where RES was added after the virus infection, showed approximately 1 log (94%) reduction of virus titer in comparison to the mock-treated cells (Fig. 3B,D), suggesting a significant decreased of the virus titer. The results of qRT-PCR also showed 7% reduction of ZIKV mRNA copy number in the pre-infection-RES-treatment regimen (Fig. 3E) while post-infection RES-treatment regimen showed 93% (1 log) reduction of virus mRNA copy number (3 F) in comparison to the mock-treated cells. These results suggested that RES exerted the antiviral effects likely after the entry of ZIKV, implying no prophylactic effects of the compound.

### RES exhibited direct virucidal effects against ZIKV particles

**Figure 4:**
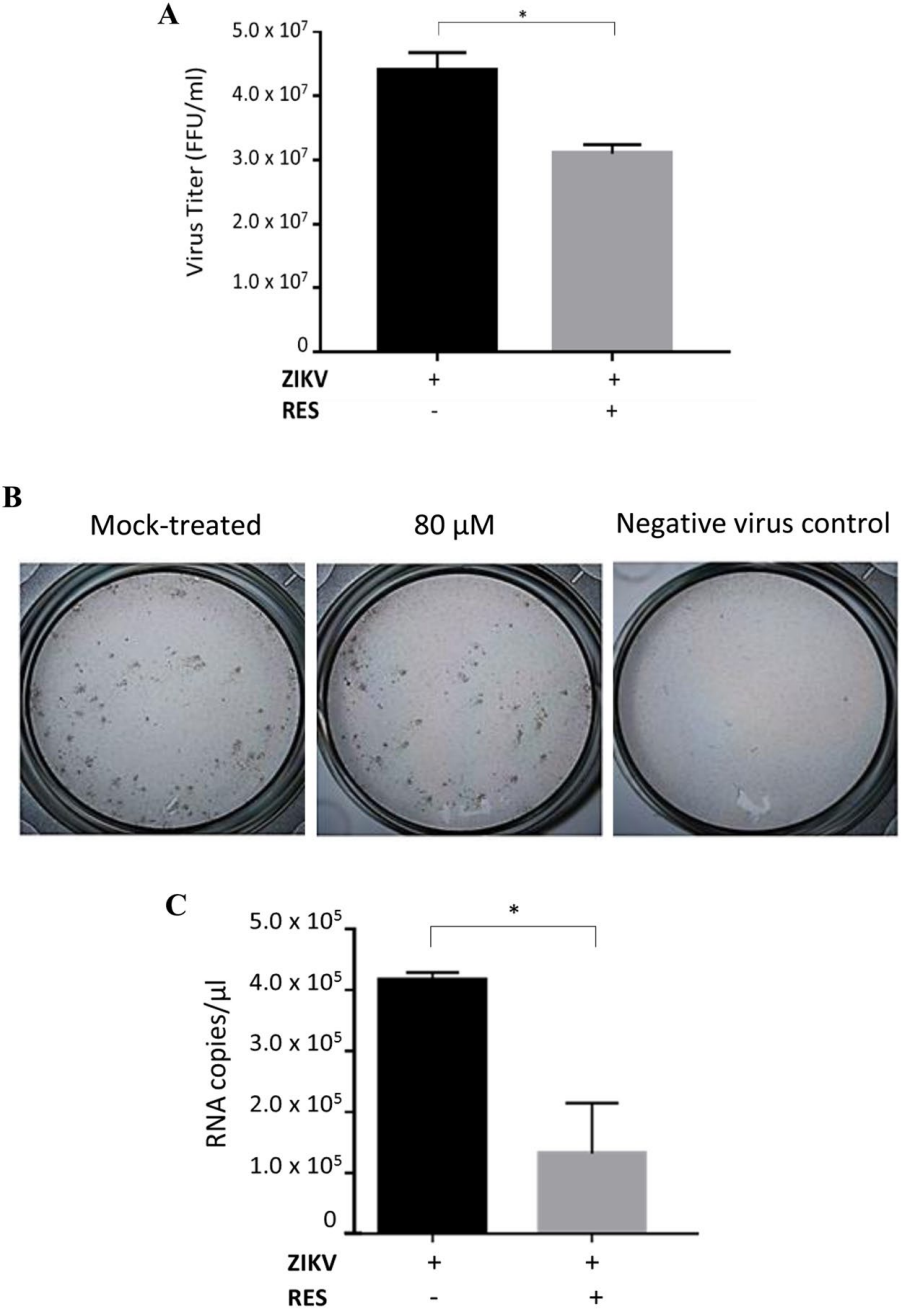
Virucidal activity of RES against extracellular ZIKV particles. Huh7 cells were infected with RES (80 µM)-pre-incubated ZIKV virus at an MOI of 1 for 48 h. The cell supernatants were collected after 48 h and virus titer was determined using focus-forming assay (**A**). Foci formed in mock- and pre-incubated RES treatment conditions after three days of incubation were shown (**B**). The mock-treated and non-infected cells were used as controls. The qRT-PCR assay was used to quantify the ZIKV mRNA copy numbers of mock-incubated and RES-pre-incubated virus conditions (**C**). Data obtained from duplicate assays were analyzed and plotted using Graph Pad Prism 7 (Graph Pad Software Inc., San Diego, CA, USA, 2016). Statistical differences between groups were as follow: *P < 0.05.

To examine whether the antiviral activity of RES could directly act on the extracellular ZIKV particles, ZIKV at an MOI of 1 was pre-incubated with 80 µM of RES for 1 h prior to infection. After 48 h, the supernatants were harvested and focus-forming assay and qRT-PCR were performed. ZIKV pre-incubated with 80 µM of RES showed 30% reduction of ZIKV titers (Fig. 4A,B) and 68% decreased of ZIKV mRNA copy number (Fig. 4C) in comparison to the non-RES-incubated ZIKV inoculum, implying a reduction in ZIKV titer. These results suggested that RES could act directly against the extracellular ZIKV particles.

### RES exhibited inhibitory effects against ZIKV by interfering with virus binding

**Figure 5:**
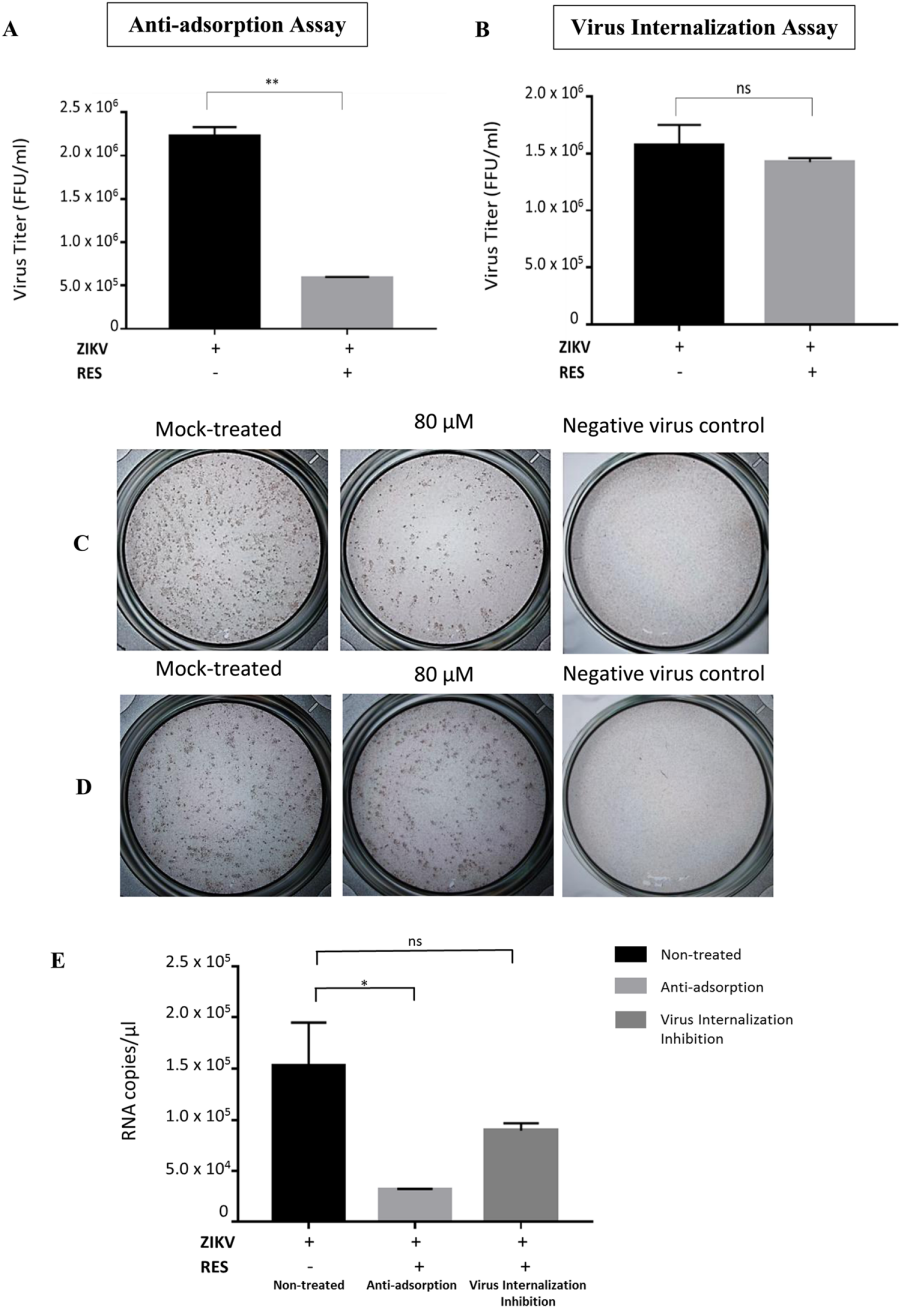
Inhibitory activities of RES against ZIKV binding to the host cell. Huh7 cells were infected with RES (80 µM)-pre-incubated ZIKV at an MOI of 1 at 4 °C for 1 h, followed by incubation for 48 h at 37 °C (anti-adsorption assay, (**A**). Huh7 cells were pre-chilled at 4 °C for 1 h and infected with ZIKV at an MOI of 1 at 4 °C for 1 h, followed by 80 µM RES-treatment for 1 h. The RES-contained medium was then replaced with overlay media and incubated for 48 h at 37 °C (virus internalization inhibition assay, (**B**). The cell supernatants were collected after 48 h and virus titer was determined using focus-forming assay. Foci formed in mock-treated, anti-adsorption assay (**C**) and virus internalization assay (**D**) conditions after three days of incubation were shown. The mock-treated and non-infected cells were used as controls. The ZIKV mRNA copy numbers were quantified using qRT-PCR (**E**). Data obtained from duplicate assays were analyzed and plotted using Graph Pad Prism 7 (Graph Pad Software Inc., San Diego, CA, USA, 2016). Statistical differences between groups were as follow: *P < 0.05, **P < 0.01.

Since RES could directly act on ZIKV particles, we examined further if RES would inhibit ZIKV binding to the host cells. Anti-adsorption assay, where RES was present during virus adsorption at 4 °C, was performed. Results obtained showed 74% (~1 log) reduction of ZIKV titers (Fig. 5A,C) and 80% reduction of ZIKV mRNA copy number (Fig. 5E) in comparison to the mock-treated cells, implying a significant decreased in virus entry in this RES-treatment regimen. In the viral internalization inhibition assay, where virus penetration was induced before the RES treatment, showed 9% reduction of ZIKV titer (Fig. 5B,D) and 40% decreased of ZIKV mRNA copy number (Fig. 5E) in the RES-treated cells in comparison to the mock-treated cells, suggesting no significant decreased of virus titer in this RES-treatment regime. Taken together, these results suggested that RES interfered with ZIKV binding, but did not directly affected viruses that were already bound to the cells.

### Virus titer analysis in the cell lysate using capture ELISA method

**Figure 6:**
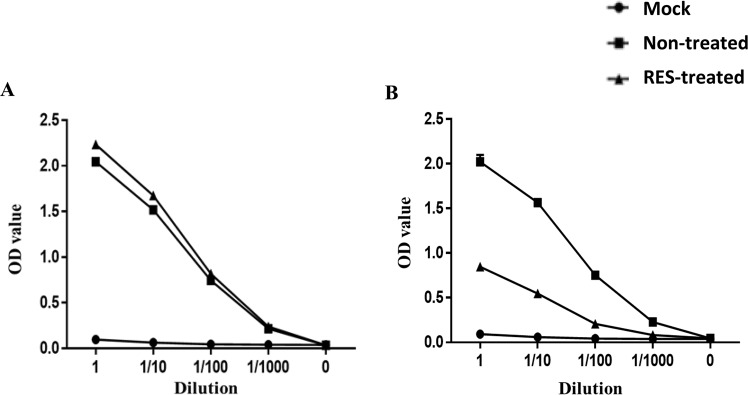
RES antiviral effects on ZIKV protein. Protein was extracted from the ZIKV-infected cells treated with 80 µM of RES. Capture ELISA for detection of ZIKV protein was performed in 96-well plate. The plate was coated with the extracted protein overnight and then incubated with increasing dilution of 4g2 antibody, followed by detection of bound antigen-antibody with peroxidase-conjugated secondary antibody (goat anti-mouse IgG) and substrate. The graphs represented different modes of RES treatments (80 µM); prior to virus infection (pre-infection) (**A**) and after virus infection (post-infection) (**B**). The absorbance value was read at 450 nm.

To further confirm the antiviral effects of RES against ZIKV, we determined the total amount of virus in the infected cell lysate using the capture ELISA developed with the anti-flavivirus envelope protein antibody (4G2). Cells were harvested from the mock-treated and RES-treated ZIKV-infected cells from the various RES-treatment conditions; pre-infection (Fig. 6A) and post-infection (Fig. 6B). Cell lysis was performed and the harvested proteins of each RES-treatment regimen were coated onto the 96-well plates. The presence of ZIKV protein was detected using the 4G2 monoclonal antibody and the absorbance value from the antigen-antibody binding was determined. In the pre-infection-RES-treatment regimen, the O.D absorbance value of RES-treated Huh7 cells was 2.23, while the mock-treated cells showed 2.05 (Fig. 6A), suggesting a non-significant reduction of ZIKV protein expression. In the post-infection-RES-treatment regimen, the RES-treated cells showed 58% reduction of the O.D absorbance value in comparison to the mock-treated cells (Fig. 6B), suggesting a significant reduction in the ZIKV protein level. These results were in concordance correlated with the earlier finding with virus titer reduction using the focus-forming assay and the ZIKV mRNA copy number determined using the qRT-PCR.

## Discussion

Plant-derived compounds as antiviral agents have been explored against many viruses^35,36^. RES, a phytoalexin isolated mainly from grapes, had been known for its anti-inflammatory properties and also as anti-oxidant agent^37,38^. It has also been shown to possess antiviral activities against a number of viruses^39–45^. Here we showed that RES possesses antiviral activities against ZIKV.

Our results suggested a dose-dependent antiviral effects of RES on ZIKV with >90% inhibition when used at just 80 µM concentration. Moreover, RES showed no-cytotoxicity effects against the cells, even when used at 100 µM concentration, which implied that the antiviral activity of RES against ZIKV is not caused by cell death. The non-cytotoxic effects of RES have been similarly documented in various other cell types, including human breast adenocarcinoma (MDA-MB 231), human cervical cancer (HeLa) and Chinese hamster lung fibroblasts (V79), where cell viability, in general, was more than 60%, even at 400 µM of RES^46^. Published human studies showed that single or multiple daily doses up to 600 mg per day of RES is safe under tested condition with no serious adverse events^47–50^. A significant reduction in virus titer following treatment with RES was only observed in the post-infection treatment, highlighting the likely possibility of non-prophylactic effects of RES against ZIKV. This finding is in agreement with the previous study^18^, where the antiviral effects of RES on DENV-infected cells were shown to be exerted through enhanced interferon-stimulated genes (ISGs) production by the host cells. The non-prophylactic effects of RES could be advantageous, as the potential over-stimulation of the host innate immune responses through activation of the ISGs^51–53^ in the uninfected cells could be mitigated.

Pre-incubation of ZIKV with 80 µM RES showed significant reduction of virus titer in the RES-treated cells in comparison to the non-treated cells. The virus titer reduction, however, was only slightly significant compared to the reduction in ZIKV mRNA copy number of the similar RES-treatment regimen. These results suggested that while the post-infection treatment was effective, the ability of RES to inhibit the free circulating ZIKV particles could have potential beneficial effects in limiting disease severity during the viraemic phase^22^. In contrary, RES showed no virucidal effects when used to treat human cytomegalovirus^54^ and adenovirus^55^ directly.

Significant reduction of ZIKV in the anti-adsorption assay implied that RES could directly inhibit virus binding to cells. Previous study has shown that RES interfered with the early phase of virus binding and entry into the host cells by inactivating the phosphorylation of the epidermal growth factor receptor (EGFR), a protein required for productive virus infection^54^. The insignificant decrease in ZIKV titer observed from the virus internalization assay in this study suggested that direct RES virus inhibition was not effective against viruses already bound to the cells. Since RES was not constantly present in the virus internalization assay, we postulated that continuous presence of RES is required for the antiviral effects against ZIKV. The continuous presence of RES could indirectly induce intracellular antiviral state against ZIKV as observed in dengue^18^. Furthermore, in an earlier study, the antiviral effects of RES were absent when added after 4 h of virus infection^54^, implying the requirement for RES to be present early and continuously to limit ZIKV infection.

While the mechanisms of how RES acted as antiviral against ZIKV is still unknown, earlier studies suggested that RES suppressed viral nuclear-cytoplasmic translocation, which subsequently affects influenza virus protein expression^39^. It also inhibits Epstein-Barr virus^40^, Herpes simplex virus^41^ and enterovirus^44^ protein expression. RES inhibits HIV replication by interfering with the reverse-transcription phase^56^ and a structure-based study suggested two RES analogs with potential antiviral activities against DENV, presumably by targeting viral RNA translation and host cellular factors^57^. More recently, we showed that RES induces antiviral responses through the regulation of a DNA-binding protein, HMGB1 and innate immune responses^18^.

In conclusion, our study suggested that RES exerts antiviral effects against ZIKV replication in a dose-dependent manner. RES could directly inactivate ZIKV through its virucidal effects, as well as interfering with virus binding to the host cells. The specific mechanisms on how RES interrupts ZIKV binding process or indirectly induces intracellular antiviral activity, however, remain to be elucidated.

## Materials and Methods

### Cells and Virus

Human hepatocellular carcinoma cells (Huh7) and African Green Monkey Kidney cells (Vero) were used in this study. Both cells were maintained in Dulbecco’s Modified Eagle Medium (DMEM, Gibco, NY) enriched with 10% of heat-inactivated fetal bovine serum (FBS, Bovogen, Australia) and L-glutamine (Gibco, NY, USA). Cells were incubated in 5% CO_2_ humidified incubator at temperature of 37 °C. The RES treatment assays were performed using Huh7 cells while the propagation of Zika virus (strain P6740) and titration of the virus were performed using Vero cells. Zika virus (ZIKV) used in this study was provided by Dr. Robert Tesh (World Reference Center for Emerging Viruses and Arboviruses, The University of Texas Medical Branch, Galveston, USA) and maintained at the Tropical Infectious Diseases Research & Education Centre (TIDREC), University of Malaya, Kuala Lumpur, Malaysia. Vero cells were infected with ZIKV and the supernatant containing viruses were harvested after seven days. Virus stock titer was determined using the focus-forming assay and stored at −80 °C until needed.

### Drug

Resveratrol (RES) used in this study was purchased from Sigma-Aldrich (St. Louis, MO). RES (10 mg/ml) was stored at −20 °C until needed and the working stock was diluted into the various concentrations (20 µM, 50 µM, 80 µM, and 100 µM) in DMEM consisting of 2% fetal bovine serum.

### Cell cytotoxicity assay

MTS assay (Promega, WI) was performed to determine the cytotoxicity levels of RES on both Huh7 and Vero cells as previously described^58^. Both cells were seeded in the 96-well plate overnight and then treated with four different concentrations of RES (20 µM, 50 µM, 80 µM, and 100 µM) in triplicates for 24 and 48 h at 37 °C in a 5% CO_2_ incubator. MTS assay was performed after each duration of treatment ended.

### Virus infection and RES treatment

#### Dose-dependent effects of RES

A monolayer of Huh7 cells was treated for 4 h with different concentrations of RES (20 µM, 50 µM, 80 µM, and 100 µM) and incubated at 37 °C in a 5% CO_2_ environment. Following the treatment, the cells were washed thrice using serum-free DMEM and then infected with ZIKV at an MOI of 1. After the infection, the cells were washed thrice with serum-free DMEM and supplemented with fresh DMEM consisting of 2% of heat-inactivated fetal bovine serum and the different RES concentrations. The cells were incubated for 48 h. Following after, the supernatants were harvested and the virus titer was determined using the focus-forming assay.

#### The effects of different RES treatment conditions

The timing of RES treatment following infection was planned into several treatment regimens. The first regimen was pre-incubation of ZIKV at an MOI of 1 with 80 µM of RES for one h prior to infection of cells. The second regimen was a pre-infection RES-treatment, where the Huh7 cells were pre-treated for 4 h with 80 µM of RES prior to virus infection. Meanwhile, the third regimen was post-infection RES-treatment, where the Huh7 cells were treated with 80 µM of RES after virus infection for 48 h. The virus titer of the mock-treated and RES-treated cells were determined using the focus-forming assay.

#### Anti-adsorption assay

ZIKV at an MOI of 1 was inoculated onto Huh7 cells in the culture medium supplemented with or without 80 µM of RES. Cells were then incubated for 1 h at 4 °C. Following after, the cells were rinsed with serum-free DMEM and each well was added with 2% DMEM-FBS, followed by incubation for 48 h at 37 °C in a 5% CO_2_ environment. Supernatants of the infected cell cultures were harvested and the virus titer was determined using the focus-forming assay.

#### Virus internalization inhibition

Huh7 cells monolayer were pre-chilled at 4 °C for 1 h. ZIKV at an MOI of 1 was inoculated onto the cells, followed by incubation for 1 h at 4 °C. The inoculum was pipetted out and the cells were washed twice with serum-free DMEM to remove the unbound virus. The cells were further incubated with and without 80 µM of RES diluted in 2% DMEM-FBS at 37 °C in a 5% CO_2_ condition for 1 h. Cells monolayers were rinsed with PBS thrice and then briefly treated with citrate buffer. After that, cells were washed three times with serum-free DMEM. Subsequently, all the wells were overlaid with 2% DMEM-FBS and incubated at 37 °C in a 5% CO_2_ environment for 48 h. The cell cultures supernatants were harvested and the virus titer was determined using the focus-forming assay.

### Focus-forming assay

ZIKV titer was determined using the foci-forming assay and shown in focus-forming units per ml (FFU/mL). The assay was performed as previously described^59^.

### Quantitative real-time polymerase chain reaction (qRT-PCR)

RNA was extracted from the harvested supernatants using the viral RNA isolation kit; QIAamp Viral RNA Mini Kit (Qiagen, CA). One-step qRT-PCR was performed using Zika virus primer sets of the polyprotein gene (Genesig® Standard Kit; Primer Design Ltd., UK). The ZIKV mRNA copy numbers were compared between the mock-treated cells and RES-treated cells. Results were presented as RNA copies per µl.

### Enzyme-linked Immunosorbent Assay (ELISA)

ZIKV protein was determined using ELISA as previously described^60^. The ZIKV protein was extracted from the Huh7 infected cells using the lysis buffer (Triton X-100, protease inhibitors, sterile cold 1xPBS). Viral protein from each sample was diluted at the desired concentration with the coating buffer. The proteins were then used to coat the 96-well plate and incubated overnight at 4 °C. The coated wells were blocked with 3% BSA for 30 mins at room temperature. Serially diluted primary antibody (Mouse anti-flavivirus envelope protein antibody [4G2] antibody from the supernatant of hybridoma cell line, American Type Culture Collection [ATCC]) was then aliquoted into the wells and incubated for 1 h at room temperature. Secondary antibody (goat anti-mouse IgG conjugated to peroxidase antibody, Merck Millipore) (1:10 000) was added into each well and incubated for another 1 h at room temperature. The reaction was developed using Tetramethylbenzidine (TMB) substrate (KPL, MA) and the absorbance reading was read at a wavelength of 450 nm, reference wavelength of 620 nm using an ELISA plate reader (Magellan, Tecan Sunrise, Australia). Each sample was assayed in triplicates and the absorbance value obtained represented the binding interaction of antibody to ZIKV antigen.

### Statistical analysis

The data obtained from the focus-forming assay, qRT-PCR, and ELISA were analyzed using Graph Pad Prism for Windows, version 7 (Graph Pad Software Inc., San Diego, CA). One-way ANOVA statistical analysis was performed, followed by Bonferroni’s multiple-comparison test. Statistically significant differences between groups were represented by *P* < 0.05. Reduction percentage of ZIKV titer and mRNA copy number (%) were calculated by comparing the virus titer and ZIKV mRNA copy number of the RES-treated and mock-treated infected cells.
